# Home electrocardiogram telemonitoring for post-acute myocardial infarction care: a randomized controlled trial

**DOI:** 10.1093/ehjopen/oeag014

**Published:** 2026-01-30

**Authors:** Meir Tabi, Zeliang Ma, Bradley Lewis, Sarah Devamani, Sabrina Rochelin, Amanda Solberg, Elaine Chiarelly, Joy Allen, Joerg Herrmann

**Affiliations:** Department of Cardiovascular Medicine, Mayo Clinic, 200 First Street SW, Rochester, MN 55905, USA; Heart Institute, H'aEmek Medical Center, Yitshak Rabin Boulevard 21, Afula 1834111, Israel; Faculty of Medicine, Technion-Israel Institute of Technology, Technion City, Haifa 3200003, Israel; Department of Cardiovascular Medicine, Mayo Clinic, 200 First Street SW, Rochester, MN 55905, USA; Division of Clinical Trials and Biostatistics, Mayo Clinic, 200 First Street SW, Rochester, MN 55905, USA; Department of Cardiovascular Medicine, Mayo Clinic, 200 First Street SW, Rochester, MN 55905, USA; Department of Cardiovascular Medicine, Mayo Clinic, 200 First Street SW, Rochester, MN 55905, USA; Department of Cardiovascular Medicine, Mayo Clinic, 200 First Street SW, Rochester, MN 55905, USA; Department of Cardiovascular Medicine, Mayo Clinic, 200 First Street SW, Rochester, MN 55905, USA; Department of Cardiovascular Medicine, Mayo Clinic, 200 First Street SW, Rochester, MN 55905, USA; Department of Cardiovascular Medicine, Mayo Clinic, 200 First Street SW, Rochester, MN 55905, USA

**Keywords:** Myocardial infarction, Health care delivery, Telemedicine

## Abstract

**Aims:**

Acute myocardial infarction (AMI) patients face a substantial risk of cardiovascular events and rehospitalization. The impact of the SmartHeart 12-lead electrocardiogram (ECG) telemedicine device on healthcare utilization has not been tested in a US randomized trial.

**Methods and results:**

Patients with AMI were randomized at discharge to standard of care without (control group) or with the SmartHeart 12-lead ECG device (intervention group). The primary endpoint was the rate of emergency department (ED) visits, hospital readmissions, and any cardiovascular testing from discharge to 90 days of follow-up. The primary endpoint was reached in 57 (59%) patients in the control group and 53 (63%) patients in the intervention group (*P* = 0.61). However, in the intervention group, only 30% of patients complied with two follow-up training SmartHeart 12-lead ECG transmissions after discharge, and only 24% used the device thereafter. Among device users, ED presentations were lower in the intervention group than in the control group (8.0% vs. 29%, *P* = 0.04). In all patients advised to present to the ED upon device use (38%), a clinically relevant cardiovascular diagnosis was made. One patient survived a ventricular fibrillation cardiac arrest as advised to present to ED urgently after device use.

**Conclusion:**

In this US-based RCT, there were no significant differences in the primary outcome in the intention-to-treat analysis. However, according to per-protocol analysis, proper use of the SmartHeart ECG device with 24/7 telemedicine support reduced inappropriate and increased appropriate ED presentations after hospitalization for AMI.

## Graphical Abstract

**Graphical Abstract:**
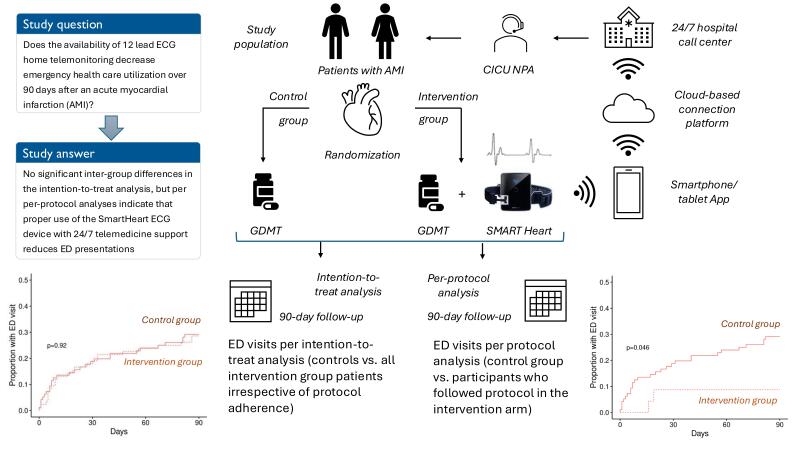

## Introduction

Survivors of acute myocardial infarction (AMI) face a significant risk of major adverse cardiovascular events (MACE), especially sudden cardiac death (SCD), recurrent myocardial infarction (MI), heart failure (HF), arrhythmias, angina, and stroke. In the United States, the 30-day mortality rate after AMI is 2–8%, and the 30-day rehospitalization rate is 17–23%, posing a substantial financial burden on the health care system at an estimated cost of $136 million.^1–5^ Innovative solutions, like telemedicine, have emerged as potential tools to improve health care utilization (decrease unnecessary and increase appropriate presentations).^6,7^

One example is the SmartHeart 12-lead electrocardiogram (ECG) device linked to SHL Telemedicine services. In retrospective studies in Europe, this setup has been shown to reduce 30-day readmission rates, decrease the time to medical assistance following symptom onset, and reduce mortality.^8–10^ Furthermore, a recent randomized clinical trial (RCT) in the United Kingdom demonstrated a significant reduction in hospital readmission rates after acute coronary syndrome (ACS) with the implementation of a telemedicine service using the SmartHeart device.^11^ Whether these findings are applicable to the USA has not been studied so far and remains a pertinent question as health care systems and cultures differ. The goal of this RCT was henceforth to define the utilization and impact of the SmartHeart 12-lead ECG device in patients discharged home after an AMI linked to a single institution health care system in the USA with 24/7 telemedicine support.

## Methods

### Study design

This is a single-site, randomized, parallel-group clinical trial, conducted at Mayo Clinic Rochester, Olmsted County, Minnesota, USA. We included patients who were admitted to the Cardiology Department or Cardiac Intensive Care Unit with the diagnosis of AMI and underwent coronary angiography. Study participants were randomized into two groups at discharge: the control group received standard medical treatment alone, while the intervention group received standard medical treatment plus a SmartHeart 12-lead ECG telemonitoring device for 90 days (see Supplementary material online, *Figure S1*). This trial was approved by the Institutional Review Board (IRB) of Mayo Clinic, and it was registered on ClinicalTrials.gov (NCT04664881). All participants have signed a written informed consent. Detailed study design is included in the Supplementary Data provided.

### Study population

Patients eligible for the study were 18 years or older with a confirmed diagnosis of AMI [both ST-Elevation Myocardial Infarction (STEMI) and non-STEMI], who underwent coronary angiography and were able to use home ECG telemonitoring with a smartphone and reliable home Wi-Fi or mobile internet for 24/7 ECG transmission. A caring family member must be available to assist with the ECG transmission if the patient is unable to perform it independently. Exclusion criteria included: being a resident of a nursing home or acute care facility, survivors of out-of-hospital cardiac arrest unrelated to ACS, secondary to a non-shockable rhythm, or with any level of neurologic damage, as well as those with uninterpretable ECGs at discharge (e.g. left bundle branch block, pacemaker, or implantable cardioverter-defibrillator with pacing dependence). Additionally, patients planned for staged percutaneous coronary intervention after the index hospitalization were also excluded.

### Study procedures

#### Enrollment and randomization

Potential participants were screened with all screening information securely recorded in an electronic log. Informed consent was obtained using a single form covering both screening and study procedures. Patients who consented underwent a detailed review of their medical history to assess eligibility. For eligible patients who declined participation, reasons were recorded in the screening log. Patients who provided informed consent were considered enrolled as of the date of consent. Baseline assessments involved a review of cardiovascular history, risk factors, and home medications. A computer-generated randomization scheme was utilized assigning each consecutive patient enrolled to one of the two groups. The randomization scheme was 1:1, and both the patient and their treating cardiologist was informed of the assigned study group.

#### Study interventions

The control group received standard medical therapy at discharge, including dual antiplatelet therapy, high-potency statin, and beta blocker as indicated, while the intervention group received the same standard therapy plus the SmartHeart 12-lead ECG device for a duration of 90 days (±7). The Food and Drug Administration-approved SmartHeart 12-lead ECG device (K113514) allows to record and transmit hospital-grade, 12-lead ECG data via a smartphone application to the monitoring centre (see Supplementary material online, *Figure S2*). Prior to being discharged from the index hospitalization, patients in the intervention group were thoroughly instructed on how to operate the device, including proper chest placement and training of ECG transmission to the study centre. They were also guided to perform two test ECG transmissions during the first 2 weeks after discharge. In order to comply, the participants received push notifications and phone calls by the study team reminding them to perform test ECGs in week 1 and 2 post discharge. Patients having difficulties with ECG transmissions in week 1 or 2 had the possibility for an additional test ECG transmission in week 4 after discharge.

#### Follow-up

Our follow-up period after the index ACS event was 3 months, based on the highest rates of complications after ACS occurring during this timeframe, especially, during the first month after ACS.^11^ Thus, during the 90-day follow-up period, patients in the intervention group were instructed to record and transmit an ECG if they experienced symptoms such as chest pain, shortness of breath, irregular heartbeats, or near fainting. Transmitted ECGs were reviewed by a dedicated nurse practitioner or physician assistant available 24/7 at the cardiac intensive care unit (CICU), supported by a CICU attending physician (see Supplementary material online, *Figure S3*). The ECG findings were assessed according to severity scale specifically developed for the study team (see Supplementary material online, *Figure S4*). Following the ECG transmission, the study team has communicated with study participants in the timeframe of 15 min since the ECG acceptance, to assess symptoms and respond to the recordings. In cases of true acute cardiac events, such as STEMI, ventricular tachycardia/ventricular fibrillation (VF), or complete atrioventricular block, patients were advised to seek immediate medical assistance, and emergency medical services would be dispatched by the CICU team to patient’s address, if necessary. Conversely, patients with reassuring ECGs and low suspicion of an acute cardiac event were advised to proceed with outpatient medical assistance, thereby potentially reducing unnecessary emergency department (ED) visits. At randomization and study completion, participants were asked to complete the MacNew quality-of-life questionnaire.

### Study outcome

The primary endpoint for comparison between control and intervention group was the event rate of one of the following: ED visits, hospital readmissions, and downstream testing modalities [transthoracic echocardiogram, cardiac stress test, coronary computed tomography (CT), or invasive coronary angiogram] at 90 days after discharge from an admission for AMI. Secondary endpoints included the individual components of the primary endpoint, incidence of ED visits, hospital readmissions (both cardiovascular and non-cardiovascular causes), and MACE, use of downstream testing modalities, total healthcare costs (quantified in US dollars) at 90 days follow-up, and quality of life scores using the MacNew questionnaire at enrollment and 90 days of follow-up. MACE was defined as cardiovascular mortality, hospitalization for myocardial infarction, unstable angina, repeat revascularization, HF, arrhythmias, or cardiac arrest.

### Statistics

The estimated event rate for the combined primary endpoint in the standard care group was 48%, with patients presenting with cardiovascular concerns potentially undergoing multiple downstream tests. To detect a 37.5% reduction in the event rate (to 30% event rate), a total sample size of 240 patients (120 per arm) would provide adequate power (80%) with a type I error rate of 0.05. This calculation accounted for the combined primary endpoint components and ensured the study was appropriately powered to detect a statistically significant difference between the intervention and control group.

The percentage of patients meeting the combined primary endpoint was compared between groups using the Chi-square test for categorical data. For continuous variables, either an independent samples *t*-test or the Wilcoxon rank-sum test was applied, depending on the distributional assumptions. Cumulative incidence curves were used to analyse the timing of the first ED visit and hospitalization. Differences between groups were tested using Gray’s test. All analyses were performed using R version 4.4.1 (R Foundation for Statistical Computing, Vienna, Austria). *P*-values below 0.05 were considered significant for the purposes of this study.

## Results

**Figure 1:**
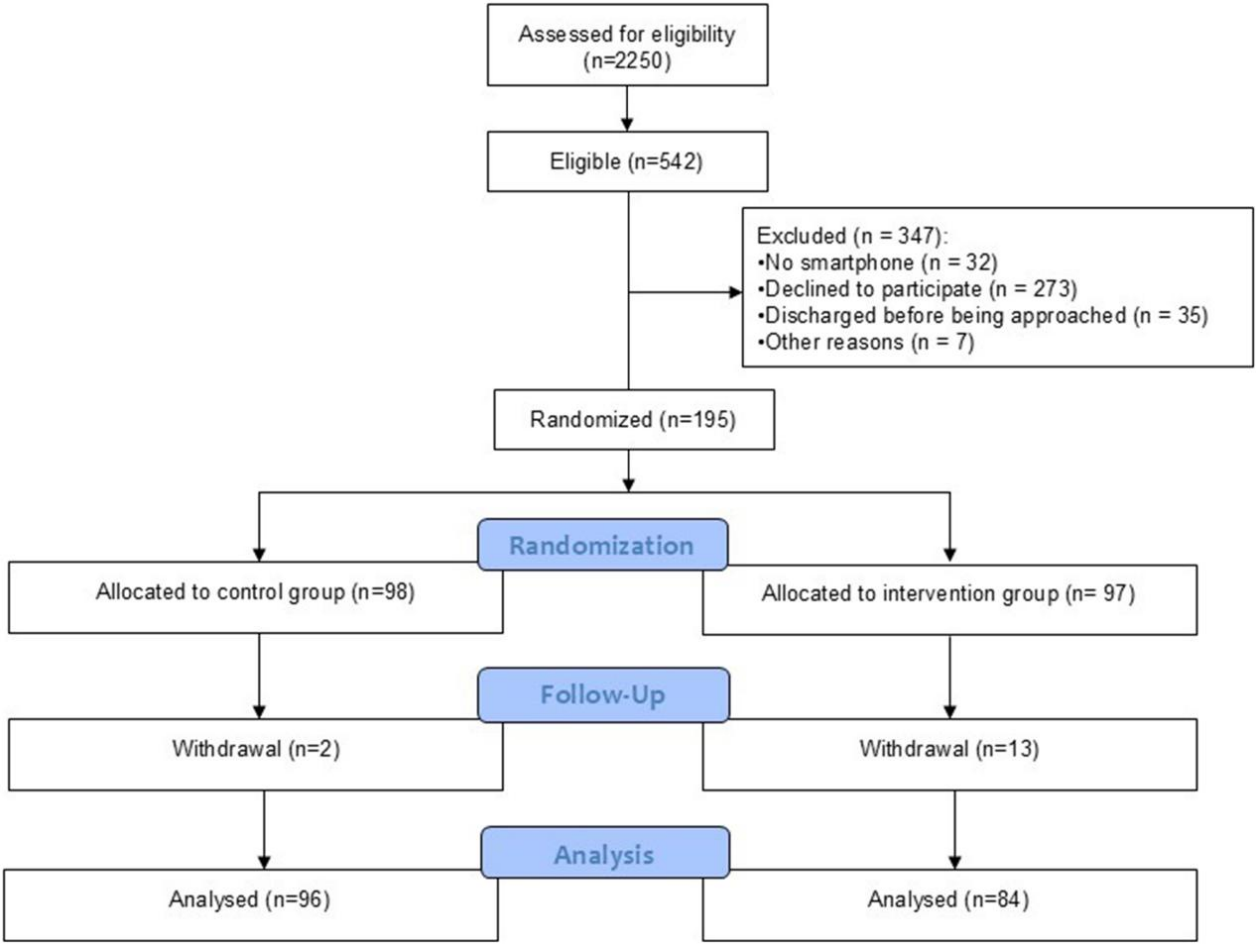
Study flowchart—final study population.

Between October 2020 and January 2024, a total of 195 participants were enrolled in the study, and a total of 180 participants completed the study follow-up and were included in the intention-to-treat analysis (*Figure 1*). Following the results of the interim analysis at 50% of patients with study completion (see Supplementary material online, *Figure S5*), the study proceeded until 75% of the planned completion rate to re-estimate the sample size, which at that point was found to far exceed the planned study volume of 240 patients, leading to the decision to stop enrollment.

Among participants who were enrolled, 96 had been randomized to the control group and 84 to the intervention group. The mean age was 61.1 (±10.8) years, and 67% of participants were male. Baseline characteristics, admission diagnosis, and hospitalization data, including the transthoracic echocardiography findings, were well balanced between the two groups, without significant differences (*Table 1*). Additionally, the medications prescribed at discharge and medications used at the end of the study were similar between the two groups (see Supplementary material online, *Table S1*).

**Table 1:** Baseline patient characteristics of the total study population and according to study groups

	Control group (*n* = 96)	Intervention group (*n* = 84)	Total (*n* = 180)	*P* value
Age	62.0 (±11.0)	60.0 (±10.4)	61.1 (±10.7)	0.22
Male gender	63 (65.6%)	58 (69.0%)	121 (67.2%)	0.62
Medical history				
Congestive heart failure	18 (18.8%)	14 (16.7%)	32 (17.8%)	0.71
Diabetes mellitus	29 (30.2%)	24 (28.6%)	53 (29.4%)	0.81
Hypertension	73 (76.0%)	55 (65.5%)	128 (71.1%)	0.11
Body mass index (kg/m^2^)	30.7 (±6.1)	30.7 (±6.2)	30.7 (±6.1)	0.64
Dyslipidaemia	63 (65.6%)	50 (59.5%)	113 (62.8%)	0.39
Family history of CAD	52 (54.2%)	49 (58.3%)	101 (56.1%)	0.57
Current smoker	22 (22.9%)	24 (28.6%)	46 (25.6%)	0.38
History of CAD	37 (38.5%)	27 (32.1%)	64 (35.6%)	0.37
Prior myocardial infarction	16 (16.7%)	13 (15.5%)	29 (16.1%)	0.82
Prior PCI	14 (14.6%)	11 (13.1%)	25 (13.9%)	0.77
Prior CABG	7 (7.3%)	5 (6.0%)	12 (6.7%)	0.71
Peripheral arterial disease	5 (5.2%)	3 (3.6%)	8 (4.4%)	0.59
Cerebrovascular disease	5 (5.2%)	4 (4.8%)	9 (5.0%)	0.90
Chronic renal disease	17 (17.7%)	9 (10.7%)	26 (14.4%)	0.18
Chronic lung disease	8 (8.3%)	8 (9.5%)	16 (8.9%)	0.77
Peptic ulcer disease	6 (6.2%)	3 (3.6%)	9 (5.0%)	0.41
Malignancy	8 (8.3%)	8 (9.5%)	16 (8.9%)	0.77
Index hospitalization data				
Presenting diagnosis				0.98
STEMI	39 (40.6%)	34 (40.5%)	73 (40.6%)	
Non-STEMI	57 (59.4%)	50 (59.5%)	107 (59.4%)	
Peak troponin (hs-cTn)	1820 (±3737)	2037 (±4246)	1923 (±3976)	0.32
Arrhythmia	2 (2.2%)	1 (1.2%)	3 (1.7%)	0.62
Transthoracic echocardiography data				
LVEF (%) mean (SD)	51.6 (±13.1)	50.7 (±14.7)	51.2 (±13.8)	0.94
LVEDD	47.9 (±13.4)	48.1 (±22.9)	48.0 (±18.5)	0.61
RVSP	25.8 (±16.7)	24.6 (±16.0)	25.2 (±16.3)	0.49
Valvular heart disease				0.12
Mild	47 (49.0%)	32 (38.1%)	79 (43.9%)	
Mild-moderate	3 (3.1%)	8 (9.5%)	11 (6.1%)	
Moderate	1 (1.0%)	2 (2.4%)	3 (1.7%)	
Diastolic dysfunction				0.34
Grade 1	19 (19.8%)	17 (20.2%)	36 (20.0%)	
Grade 2	1 (1.0%)	1 (1.2%)	2 (1.1%)	
Left atrial volume index	27.4 (±13.4)	24.8 (±12.4)	26.2 (±13.0)	0.74

Data are presented as mean (±SD) or *N* (%).

CAD, coronary artery disease; CABG, coronary artery bypass grafting; LVEF, left ventricular ejection fraction; LVEDD, left ventricular end-diastolic diameter; PCI, percutaneous coronary intervention; RVSP, right ventricular systolic pressure; STEMI, ST-segment elevation myocardial infarction.

**Figure 2:**
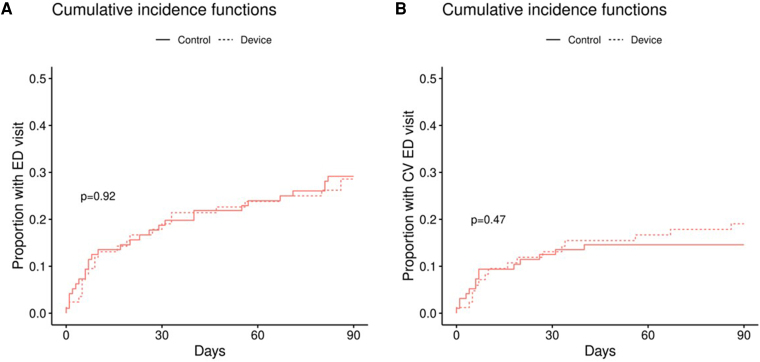
The proportion of (A) general ED visits and (B) CV-related ED visits over 90 days follow-up per intention-to-treat analysis.

**Figure 3:**
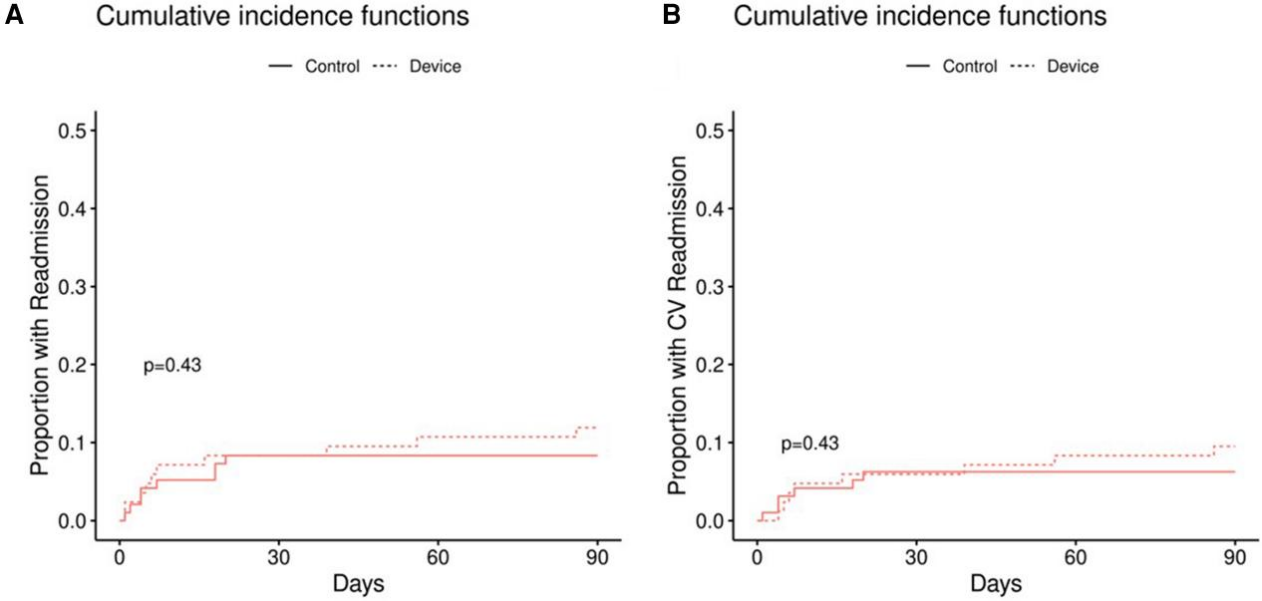
The proportion of (A) general hospitalization and (B) CV-related hospitalization over 90 days follow-up per intention-to-treat analysis.

Over the 90-day follow-up period, the primary endpoint was reached in 57 patients (59.4%) in the control group and 53 patients (63.1%) in the intervention group (*P* = 0.61). Detailed outcomes are summarized in *Table 2*. Comparing the control group vs. the intervention group, there were no significant differences in ED visits (29.2% vs. 28.6%, *P* = 0.93), including cardiovascular (CV)-related ED visits (14.6% vs. 19%, *P* = 0.42) (*Figure 2A and B*). The total number of ED visits for CV-related reasons did not differ between the control and the intervention group (*P* = 0.26). General and CV-related hospitalizations were also similar between the groups (*Figure 3A and B*). Furthermore, there were no significant differences between the two groups in terms of downstream testing, except for a trend in utilization of echocardiogram, which was performed in 12 patients (12.5%) in the control group and 4 patients (4.8%) in the intervention group (*P* = 0.07). There was no significant difference in the incidence of MACE between the two groups, with two cases (2.1%) in the control group and six cases (7.1%) in the intervention group (only one patient used the device and was appropriately triaged, *P* = 0.10). There were a total of 6 (3.3%) patients who died over the follow-up period, three in the control group (one with an unknown and two with a cardiac cause of death), and three in the intervention group (one with an unknown and two with a cardiac cause of death, none with device use, *P* = 0.87).

**Table 2:** Primary and secondary end-point events

	Control group (*n* = 96)	Intervention group (*n* = 84)	Total (*n* = 180)	*P* value
ED visit	28 (29.2%)	24 (28.6%)	52 (28.9%)	0.930
Number of ED visits				0.425
0	68 (70.8%)	60 (71.4%)	128 (71.1%)	
1	19 (19.8%)	17 (20.2%)	36 (20.0%)	
2	5 (5.2%)	6 (7.1%)	11 (6.1%)	
3	2 (2.1%)	0 (0.0%)	2 (1.1%)	
4	2 (2.1%)	0 (0.0%)	2 (1.1%)	
8	0 (0.0%)	1 (1.2%)	1 (0.6%)	
ED visit—CV cause	14 (14.6%)	16 (19.0%)	30 (16.7%)	0.423
Number of ED visits—CV				0.262
0	82 (85.4%)	68 (81.0%)	150 (83.3%)	
1	10 (10.4%)	14 (16.7%)	24 (13.3%)	
2	4 (4.2%)	1 (1.2%)	5 (2.8%)	
8	0 (0.0%)	1 (1.2%)	1 (0.6%)	
Hospitalization	8 (8.3%)	10 (11.9%)	18 (10.0%)	0.426
Number of hospitalizations				0.832
0	87 (90.6%)	74 (88.1%)	161 (89.4%)	
1	6 (6.2%)	8 (9.5%)	14 (7.8%)	
2	2 (2.1%)	1 (1.2%)	3 (1.7%)	
3	1 (1.0%)	1 (1.2%)	2 (1.1%)	
CV hospitalization	6 (6.2%)	8 (9.5%)	14 (7.8%)	0.413
Number of CV hospitalizations				0.167
0	89 (92.7%)	76 (90.5%)	165 (91.7%)	
1	4 (4.2%)	7 (8.3%)	11 (6.1%)	
2	3 (3.1%)	0 (0.0%)	3 (1.7%)	
3	0 (0.0%)	1 (1.2%)	1 (0.6%)	
Cardiac-related tests completed during follow-up				
Electrocardiogram	47 (49.0%)	50 (59.5%)	97 (53.9%)	0.156
Echocardiogram	12 (12.5%)	4 (4.8%)	16 (8.9%)	0.069
Stress test	11 (11.5%)	6 (7.1%)	17 (9.4%)	0.323
Angiography	3 (3.1%)	7 (8.3%)	10 (5.6%)	0.128
Cardiac CT	0 (0.0%)	2 (2.4%)	2 (1.1%)	0.128
MACE	2 (2.1%)	6 (7.1%)	8 (4.4%)	0.100
MacNew QoL Baseline	5.428 (0.946)	5.441 (1.000)	5.434 (0.969)	0.789
MacNew QoL at End	6.117 (0.701)	6.120 (0.799)	6.118 (0.743)	0.953
Change in MacNew QoL	0.648 (0.803)	0.507 (0.909)	0.587 (0.850)	0.565

Data are presented as mean (±SD) or *N* (%).

CV, cardiovascular; ED, emergency department; MACE, major adverse cardiovascular events; MacNew QoL, MacNew Quality of Life.

There was no significant difference in total treatment-related healthcare costs, during the follow-up period, between the control and intervention groups (*P* = 0.617). The change between the baseline and 90-day score of MacNew quality of life questionnaire did not differ significantly; the control group had a mean change of 0.648 [95% CI: (0.418, 0.878)], while the intervention group had a mean change of 0.507 [95% CI: (0.222, 0.792)], (*P* = 0.565).

**Figure 4:**
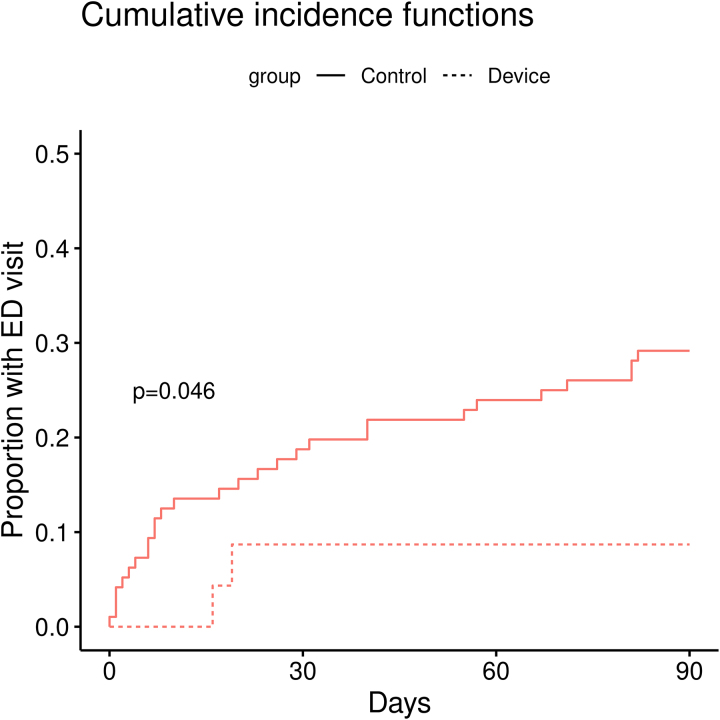
The proportion of general ED visits over 90 days follow-up according to per-protocol analysis.

Overall, the utilization of the SmartHeart device was low. In the intervention group, only 25 patients (30%) completed the two scheduled follow-up training calls with ECG transmissions after discharge, and only 21 patients (24.4%) went on to use the device thereafter. The recommendations following true clinical ECG transmissions (*n* = 34) were as follows: 40.6% were advised to go to the ED, 31.3% were instructed to consult their primary care provider, and 28.1% received reassurance without further immediate action. Of the 24 CV-related ED visits in the intervention group, 6 (25%) could have been avoided if the SmartHeart device had been used with review/consultation of the team. Indeed, when the analysis was restricted to patients who used the SmartHeart device (those who completed the two testing phases) and ED visits were only counted if the device was used prior to presentation (per protocol analysis), the overall rate of ED presentations differed significantly between the groups: 28 out of 96 patients (28.6%) in the control group compared to 2 out of 25 patients (8.0%) in the intervention group (log-rank *P* = 0.046) (*Figure 4*). All patients who were advised to go to the ED upon per protocol engagement were appropriately advised to do: 10 cases with chest pain [3 of which were non-STEMI], 2 cases of atrial fibrillation, and 1 case of VF. One of these cases resulted in an aborted SCD (see Supplementary material online, *Figure S6*). Comparing patients in the intervention group who were compliant with device use vs. those who were not did not reveal any significant differences other than a higher prevalence of malignancy, both active and in remission, in the compliant device subgroup (see Supplementary material online, *Table S2*).

A post-trial survey among device users revealed that about one-third had technical issues that prevented them from using the device, mainly correct device placement and network connection (see Supplementary material online, *Table S3*). Twenty percent of patients were not fully aware of the specific requirements and purpose of the test calls. Twenty-five percent forgot about the test calls, and 5.8% felt anxious about them. Among users, the experience was universally positive.

## Discussion

This RCT investigated the utilization and impact of a home 12-lead ECG device in post AMI patients in the United States. Utilization rate of the SmartHeart 12-lead ECG device was low, leading to no significant differences in the primary outcome between the two study groups in the intention-to-treat analysis. In the per-protocol analysis, however, proper use of the SmartHeart ECG device with telemedicine support reduced inappropriate and increased appropriate ED presentations after hospitalization for AMI.

Studies have noted increasing non-adherence to medications in the USA, and telemedicine is one of the proposed solution.^12–14^ As concluded in an American Heart Association Statement on ‘Harnessing Mobile Health Technology for Secondary Cardiovascular Disease Prevention in Older Adults’, improvements in health behaviours can be observed by use of technology, particularly if combined with a short message service (e.g. texting).^15^ This point, the importance of adjusting to the proper patient population, and a comprehensive team with 24/7 availability was highlighted in the Telemedical Interventional Management in patients with Heart Failure (TIM-HF) trial. This study randomly assigned chronic HF patients to usual care or remote telemedical management (RTM) with portable devices for ECG, blood pressure, and body weight measurements connected to a personal digital assistant that sent automated encrypted transmission via cell phones to the telemedical centres in Germany. Of the patients assigned to RTM, 287 (81%) were at least 70% compliant with daily data transfers for more than 30 days. Compared with usual care, RTM had no significant effect on all-cause mortality, cardiovascular death, or HF hospitalization over a median follow-up of 26 months.^16^ A post hoc analysis, however, helped to identify a subpopulation in which RTM could be successful. This led to the TIM-HF2 trial, which randomized patients who were free of depression and admitted for HF within the preceding 12 months to a comprehensive RTM care team approach or usual care. The percentage of days lost due to unplanned cardiovascular hospital admissions and all-cause death was significantly reduced by 20% in the RTM group.^17^ Of note, this effect was lost once patients stopped the RTM programme.^18^ Akin to these findings, a retrospective cohort study of patients with a recent HF admission and at high risk for rehospitalization found that those who participated in a comprehensive telehealth programme (including daily remote telemonitoring of HF signs/symptoms and regular individualized telecoaching sessions) had a significantly lower number of re-hospitalizations for HF or any cause, and all-cause mortality compared to propensity matched HF patients not participating in this programme.^19^

Akin to HF, studies have started to evaluate the use of telehealth in patients after an AMI.^20,21^ Retrospective studies on the impact of SHL Telemedicine, in particular, showed considerable promise for reducing 30-day readmission rates of post-AMI patients as well as shortening the interval between symptom onset and call for medical assistance, and compared to a national comparator group, patients using this device had lower post-AMI mortality.^8–10^ The recent randomized controlled TELE-ACS trial, which was conducted in the UK, showed significant benefits with the use of the SmartHeart device in post-AMI patients, including reduced hospital readmissions, ED visits, and improved symptom management over 6 months.^11^ There are several important differences to be noted between this and our study. Firstly, in the TELE-ACS study, 100% of the participants made at least one training call to the research team and 50% of the patients used the device thereafter compared to 30% and 24% in our study, respectively. The lower engagement in our study was also evident during the enrollment process. Notably, in TELE-ACS, 337 out of 372 eligible patients (90.6%) were accrued and randomized compared to only 273 out of 542 eligible patients (50.4%) in our study. These data allude to important differences in health care and research mentalities across different regions in the world and underscore that any observation made in one part of the world needs to be retested in the specific health care system it is used in. Patients in the USA rely on ED services more heavily than patients in other countries. This has been shown for overall and mental health visits.^22,23^ For many in the USA, the ED has become not their last but their first resort and a default place for health care. Participation in this study did not change this. Indeed, some participants in this study who were given the device questioned why they would do the home ECG and wait for the call back when there is a chance to present to the ED right away, where they can be comprehensively care for.

Secondly, the intervention design of TELE-ACS incorporated multiple monitoring tools, including ECG, blood pressure, and pulse oximetry, alongside cardiologist-led remote consultations. In contrast, our study entailed the SmartHeart ECG device use with immediate review and call back by a nurse practitioner in the CICU, trained in ECG interpretation and patient assessment, along with cardiologist backup. Thirdly, the endpoints differed between the two studies: time to first hospital admission over a 6-month follow-up in TELE-ACS vs. a 90-day combined event rate (either ED visit, hospitalization, or cardiac imaging/CAD testing) in our study. In direct comparison of 3-month rate of ED visit and hospitalization (information as provided in the TELE-ACS), the time-to-readmission curves already showed impressive diversion for the two groups. The difference was not that extensive for the curves reflecting time-to-ED presentation. Any such inter-group differences were not seen in our study.

An important aspect of our study is that the two main goals of the intervention go in opposite directions, i.e. reduction of unnecessary visits and testing on the one hand and an increase in early detection and intervention on the other hand, but ultimately converge on the same endpoint. An analysis of the transmissions and outcomes in the intervention group in our study attests that an ED presentation can be safely deferred in the majority of cases (60% herein). These findings confirm the device’s value in optimizing healthcare resource utilization when properly trained and implemented. Conversely, in all patients advised to present to the ED, a clinically relevant diagnosis was made and all of them were admitted for CV reasons. In fact, one life was saved due to the proper use of the intervention and referral to the local ED where the patient coded upon presentation (see Supplementary material online, *Figure S6*). From an endpoint perspective, this event is counted against the intervention; however, from a patient-centered perspective, it should be considered in favor of it.

The major factor undermining the primary endpoint was compliance of patients in the device group. Patients were trained how to place the device and how to log in and connect with the App and had to demonstrate proficiency to qualify for the study. Furthermore, all patients had to complete a successful ECG transmission before discharge, the quality of which was reviewed. They were informed that this was to be repeated twice after discharge, and this adherence metric was prospectively defined and monitored. Text message reminders for post-discharge ECG test transmissions were developed as well. However, patients may have viewed these as optional and the device as a possible backup only. A post-study survey was conducted to gain more insight, and technical and psychological barriers were identified, which might be informative for the use of home monitoring devices in general. Any such technology needs to be user-friendly (e.g. smartphone or tablet interface with easy prompts, check-ins, and reminders), simple to use, and supported by a service line. Training of and follow-up with household members or caregivers might be helpful as well, especially if any such technology is used under very stressful circumstances such as those generating fear of suffering an AMI.

### Limitations

Several limitations of our study should be acknowledged. Firstly, the low compliance rate in the intervention group hindered the ability to detect significant differences. The trial was stopped early in view of a futility analysis and remained underpowered for the primary endpoint. Secondly, the single-centre design restricts the generalizability of the findings to other settings or populations. A repeat trial should involve multiple centres and implement what was learned herein. Reimbursement models, digital literacy, and ED-use culture in the USA differ from the UK/Europe, which is of consideration in this context and may explain the lower engagement in this trial. Thirdly, although the incidence of ED visits and hospitalizations is the highest during the first 3 months following AMI, a substantial number of ED visits and hospitalizations continue to occur beyond this period. As such, the 90-day follow-up in our study may not fully capture the long-term potential and benefits of the device in reducing healthcare utilization and improving patient outcomes. Extending the monitoring and follow-up period could provide a more comprehensive assessment of the device's impact on patient care and resource utilization over time. Lastly, trials that test an intervention that has two opposite goals, both of which are beneficial (e.g. decreasing inappropriate presentations and increasing appropriate presentations) yet equally count towards the primary endpoint (e.g. total ED presentations), face limitations to prove statistically and clinically important differences and benefit from a suitable pre-defined net benefit approach.

## Conclusion

This US-based RCT did not show significant differences in the primary outcome in the intention-to-treat analysis, mainly due to a low utilization rate of the SmartHeart ECG device. However, proper use of the SmartHeart ECG device with 24/7 telemedicine support reduced inappropriate and increased appropriate ED presentations after hospitalization for AMI. These findings suggest that improving patient engagement and compliance with telemonitoring devices is essential to harness their full potential in post-AMI care.

## Supplementary Material

oeag014_Supplementary_Data

## Data Availability

The datasets generated and/or analyzed during this study are available from the corresponding author on reasonable request. The study protocol and other related documents can also be made available to others on request. The corresponding time frame shall be with publication and can be shared as an electronic file after explicit approval of the study investigators and a signed data usage agreement between the participating institutions.
