# Supplementary material for: Home electrocardiogram telemonitoring for post-acute myocardial infarction care: a randomized controlled trial
Source: Eur Heart J Open. 2026 Jan 30;6(1):oeag014. doi: 10.1093/ehjopen/oeag014 (PMC12958020; doi:10.1093/ehjopen/oeag014)
Supplement: oeag014_Supplementary_Data [file oeag014_Supplementary_Data.docx]

SUPPLEMENTAL STUDY DATA

**Home ECG Telemonitoring for Post-Acute Myocardial Infarction Care: A Randomized Controlled Trial**

Meir Tabi, M.D.^1,2^*; Mark Zeliang, M.D.^1*^; Bradly Lewis,^3^; Sarah Devamani,^1^;

Sabrina Rochelin,^1^; Amanda Solberg,^1^; Elaine Chiraelly,^1^; Joy Allen,^1^; Joerg Herrmann, M.D.^1^

^1^ Department of Cardiovascular Medicine, Mayo Clinic, Rochester, MN, USA

^2^ Heart Institute, H'aEmek Medical Center, Afula, Israel. Faculty of Medicine, Technion-Israel Institute of Technology, Haifa, Israel.

^3^ Division of Clinical Trials and Biostatistics, Mayo Clinic, Rochester, MN, USA

* These authors contributed equally to this work.

**Supplemental Content**

**Supplemental Table 1: Medication used**

**Supplemental Table 2.** **Patient characteristics in the device group by compliance status.**

**Supplemental Table 3: Device user survey results**

**Supplemental Figure 1: Intervention device and app used**

**Supplemental Figure 2: Decision making tool for study team**

**Supplemental Figure 3: Study workflow diagram**

**Supplemental Figure 4: Study outline**

**Supplemental Figure 5: Interim analysis results**

**Supplemental Figure 6: Study participant case example**

**Supplemental Table 1.** Medication use at discharge and at the end of the study. Data presented as N (%).

|  | At discharge | | | At the end of the study | | |
| --- | --- | --- | --- | --- | --- | --- |
| Medication | **Control (N=96)** | **Intervention (N=84)** | **p value** | **Control (N=96)** | **Intervention (N=84)** | **p value** |
| ACEI | 46(47.9%) | 44 (52.4%) | 0.550 | 48 (50.0%) | 41 (48.8%) | 0.873 |
| ARB | 13(14.4%) | 15 (19.0%) | 0.428 | 21 (22.3%) | 15 (18.8%) | 0.560 |
| Beta Blocker | 82 (85.4%) | 71 (84.5%) | 0.867 | 81 (86.2%) | 72 (87.8%) | 0.748 |
| Statin (high potency) | 86 (89.6%) | 79 (94.0%) | 0.280 | 86 (89.6%) | 77 (91.7%) | 0.633 |
| Aspirin (low dose) | 82 (85.4%) | 74 (88.1%) | 0.598 | 78 (82.1%) | 68 (81.9%) | 0.975 |
| Clopidogrel | 81 (84.4%) | 72 (85.7%) | 0.802 | 79 (82.3%) | 73 (86.9%) | 0.394 |
| Ticagrelor | 1 (1.0%) | 0 (0.0%) | 0.348 | 2 (2.1%) | 0 (0.0%) | 0.195 |
| Diuretics | 12 (12.5%) | 9 (10.7%) | 0.710 | 14 (14.7%) | 10 (12.2%) | 0.622 |

ACEI, Angiotensin-Converting Enzyme Inhibitor; ARB, Angiotensin Receptor Blocker.

**Supplemental Table 2.** Patient characteristics in the device group stratified by compliance status. Data presented as Mean (±SD) or N (%).

|  | Non-compliant (N=59) | Compliant (N=25) | p value |
| --- | --- | --- | --- |
| Age | 59.2 (±10.7) | 62.0 (±9.6) | 0.24 |
| Male Gender | 40 (67.8%) | 18 (72.0%) | 0.70 |
| Past Medical History | | | |
| Congestive Heart Failure | 10 (16.9%) | 4 (16.0%) | 0.92 |
| Diabetes Mellitus | 14 (23.7%) | 10 (40.0%) | 0.13 |
|  |  |  |  |
| Hypertension | 38 (64.4%) | 17 (68.0%) | 0.75 |
| Body Mass Index (kg/m^2^) | 31.2 (±6.41) | 29.7 (±5.50) | 0.42 |
| Dyslipidemia | 35 (59.3%) | 15 (60.0%) | 0.95 |
| Family History of CAD | 32 (54.2%) | 17 (68.0%) | 0.24 |
| Current Smoker | 17 (28.8%) | 7 (28.0%) | 0.94 |
| History of CAD | 20 (33.9%) | 7 (28.0%) | 0.60 |
| Prior Myocardial Infarction | 9 (15.3%) | 4 (16.0%) | 0.93 |
| Prior PCI | 6 (10.2%) | 5 (20.0%) | 0.22 |
| Prior CABG | 4 (6.8%) | 1 (4.0%) | 0.62 |
| Peripheral Arterial Disease | 2 (3.4%) | 1 (4.0%) | 0.89 |
| Cerebrovascular Disease | 2 (3.4%) | 2 (8.3%) | 0.34 |
| Chronic Renal Disease | 5 (8.5%) | 4 (16.0%) | 0.31 |
| Chronic Lung Disease | 7 (11.9%) | 1 (4.0%) | 0.26 |
| Peptic Ulcer Disease | 3 (5.1%) | 0 (0%) | 0.25 |
| Malignancy | 1 (1.7%) | 7 (28.0%) | <0.001 |
| -in remission | 1 (1.7%) | 3 (12.0%) | 0.04 |
| -active | 0 (0%) | 4 (16.0%) | 0.002 |
| Index Hospitalization Data | | | |
| Presenting Diagnosis |  |  | 0.67 |
| STEMI | 23 (39.0%) | 11 (44.0%) |  |
| Non-STEMI | 36 (61.0%) | 14 (56.0%) |  |
| Peak Troponin (hs-cTn) | 2231 (±4462) | 1582 (±3736) | 0.45 |
| Arrhythmia | 1 (1.7%) | 0 (0%) | 0.52 |
| Transthoracic Echocardiography Data | | | |
| LVEF (%) Mean (SD) | 49.4 (±15.0) | 53.6 (±14.1) | 0.10 |
| LVEDD | 47.5 (±23.9) | 49.8 (±20.7) | 0.82 |
| RVSP | 23.5 (±17.7) | 27.0 (±12.2) | 0.15 |
| Valvular Heart Disease |  |  | 0.45 |
| Mild | 22 (37.3%) | 7 (28.0%) |  |
| Mild-Moderate | 5 (8.5%) | 3 (12.0%) |  |
| Moderate | 1 (1.7%) | 1 (4.0%) |  |
| Diastolic Dysfunction |  |  | 0.19 |
| Grade 1 | 9 (15.3%) | 8 (32.0%) |  |
| Grade 2 | 1 (1.7%) | 0 (0%) |  |
| Left Atrial Volume Index | 25.8 (±11.9) | 22.6 (±13.7) | 0.74 |

CAD, Coronary Artery Disease; STEMI, ST-segment Elevation Myocardial Infarction; PCI, Percutaneous Coronary Intervention; CABG, Coronary Artery Bypass Grafting; LVEF, Left Ventricular Ejection Fraction; LVEDD, Left Ventricular End-Diastolic Diameter; RVSP, Right Ventricular Systolic Pressure

**Supplemental Table 3.** Device user survey results

|  | Unclear on how to start the device | Unable to correctly position the device | Did not know how to send the ECG | Device malfunction (e.g., error messages, power failure) | Unable to connect the device to a phone or network | Other technical issues | No | Participant Comments |
| --- | --- | --- | --- | --- | --- | --- | --- | --- |
| Q1.1 - Did you encounter any of the following technical issues that prevented you from completing the device tests | 0.00% | 17.70% | 0.00% | 0.00% | 8.80% | 5.90% | 79.40% | Unreasonable to do what they wanted  Patient said they are not computer savvy. They were unable to complete tasks with instructions over the phone. |
|  | Yes | No |  |  |  |  |  |  |
| Q1.2 - Do you feel the training you received was sufficient to help you complete the tests? | 97.10% | 2.90% |  |  |  |  |  |  |
|  | Fully aware | Partially aware | Not aware |  |  |  |  |  |
| Q1.3 -  Were you aware of the specific requirements and the purpose of the device tests? | 79.40% | 20.60% | 0.00% |  |  |  |  |  |
|  | It was not mandatory | Did not believe that the tests were necessary for my health | Found the process complicated or inconvenient | Forgot to complete the tests | Felt anxious or uneasy about using the device | Other psychological reasons | Not Applicable |  |
| Q1.4 - What were the main reasons for not completing the tests? | 2.90% | 2.90% | 5.90% | 23.50% | 2.90% | 2.90% | 61.80% | "That's what I told them when they called. I didn't see a need to use it when I didn't have any symptoms." |
|  | Did not receive reminders from the study team | Too busy to complete the tests | Lack of support from family or friends | Inconvenient testing environment (e.g., no access to power or network) | Could not locate the device in time | Other external factors | No |  |
| Q1.5 - Were there any external factors that affected your ability to complete the tests? | 11.80% | 11.80% | 2.90% | 0.00% | 0.00% | 5.9 | 70.60% | "Busy travelling for work and did not get the time"  A little busy after getting back to work  Patient's device was left in truck, and truck got broken into. Device was stolen, and patient never used device  Patient mentioned they live alone and did not have family present to help |
|  | Clearer instructions for operation | Improved ease of use | More frequent reminders (e.g., SMS or phone call notifications) | Enhanced technical support and service | Improved portability and accessibility of the device | Other suggestions | None |  |
| Q1.6 - What aspects of the device do you think could be improved to help you complete the tests more easily? | 0.00% | 0.00% | 26.50% | 11.80% | 0.00% | 11.80% | 61.90% | Patient feels it was cumbersome to put on if he was having symptoms of a heart attack. They would have preferred a hand held device, than a strapped device  "The purpose of the device and study is great but hopefully technology has improved, where patients do not have to use the strap any longer, like they have the smart watches that detect ECG"  Patient understands that the stretchable bands are for different size bodies but suggested that Velcro band would have helped keep the device in place |
|  | Yes, please go to the next question | No, please go to question #4 | |  |  |  |  |  |
| Q2.1 - Did you use the device during symptoms (not including test uses)? | 8.80% | 91.20% |  |  |  |  |  |  |
|  | Positive, could connect with the medical team and receive proper care. Please go to question #8 | Not positive | Did not use device during symptoms | Did not have symptoms | |  |  |  |
| Q2.2 - What was your experience using the device during symptoms? | 100.00% | 0.00% | 0.00% | 0.00% |  |  |  | "Very positive - within minutes I received a call from St. Mary's nurse. They told me it was serious and I went to my local ED, where they had already communicated to. I am extremely happy, it saved my life. I wouldn't be talking to you, if not for the device." |
|  | Could not connect the device | Did not receive a call back from the medical team | Review with the medical team was not helpful | Other reasons | Does not apply | |  |  |
| Q2.3 - If your experience was not positive, what were the main reasons? | 0.00% | 0.00% | 0.00% | 0.00% | 100.00% |  |  |  |
|  | Felt that seeking medical care directly was faster or more reliable | Forgot the device's function or how to operate it | Device was not nearby or could not be found | Believed the device could not help with my condition | Felt anxious or uneasy about using the device | Other reasons | Not applicable |  |
| Q2.4 - What were the main reasons for not using the device during symptoms? | 0.00% | 2.90% | 0.00% | 0.00% | 0.00% | 0.00% | 97.10% |  |
|  | Could not locate the device during symptom onset | Lack of support from family or friends to operate the device | Inadequate access to power or network connectivity | Other external factors (please specify) | No |  |  |  |
| Q2.5 - Did any of the following external factors prevent you from using the device during symptoms? | 0.00% | 2.90% | 0.00% | 2.90% | 93.80% |  |  | Patient's device was left in truck, and truck got broken into. Device was stolen, and patient never used device |
|  | Fully aware | Partially aware | Not aware |  |  |  |  |  |
| Q2.6 - Were you aware of the requirements and the purpose of using the device during symptoms? | 91.20% | 5.90% | 2.90% |  |  |  |  |  |
|  | Yes | No, please specify areas for improvement | | |  |  |  |  |
| Q2.7 - Do you feel the training you received was sufficient to help you use the device? | 91.20% | 8.80% |  |  |  |  |  |  |
|  | Clearer usage instructions | Improved portability and accessibility | Real-time technical support | Other suggestions | None |  |  |  |
| Q2.8 - What aspects of the device or process do you think could be improved to encourage use during symptoms? | 0.00% | 2.90% | 5.90% | 23.50% | 67.70% |  |  | Staff who is training the patient should be trained well. They were new and were not confident  Patient was very happy with the device, and felt confident, if they were to have a heart attack  "No, I wouldn't change anything. It was a good experience, I felt at ease having the device with me, in case it was actual symptoms or mentally thought it was."  None, it can save lives and can really be useful if patients use it  Help during set up process  "Patient should be proactive, I was not"  "Everything was explained very clearly, but making sure that every patient has the opportunity to use the device would be my suggestion".  Very straightforward, nothing to do differently.  Patient thinks it's an ideal and wonderful device if they were more computer savvy. They prefer hands on learning and wanted more time with device in-person. |

**Supplemental Figure 1.** Study outline


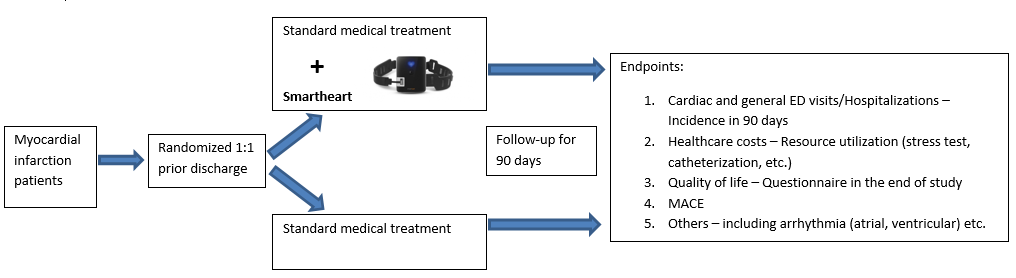


**Supplemental Figure 2.** Intervention device and app used, provided by SHL Telemedicine**.**


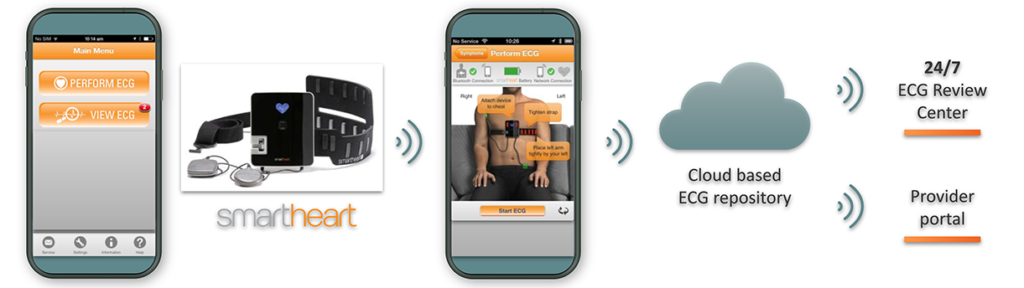


**Supplemental Figure 3.** Study workflow diagram


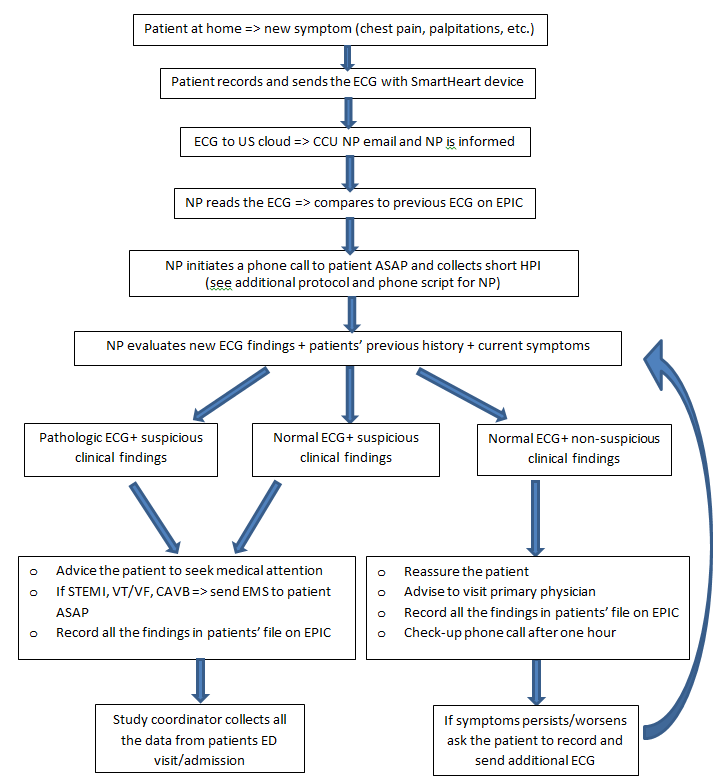


**Supplemental Figure 4.** Decision making tool for study team

**Supplemental Figure 5.** Interim analysis results

**A**

**B**

*

*

Summary of the interim analysis, based on intention-to-treat (panel A) and stratified by compliance with the study instructions (call in for test calls and use of the device as needed in follow-up, panel B). Cardiovascular (CV) visits to the emergency department (ED) and hospitalization showed a trend towards reduction in the compliant device group (*p=0.075).

**Supplemental Figure 6.** Case example

**A**

**B**

Example of a 60 year-old male who enrolled into the HELP-ME trial after being hospitalized with a myocardial infarction with non-obstructed coronary arteries (MINOCA). He was randomized to the device arm, was compliant with device use and transmitted an ECG after he developed chest pain and diaphoresis while walking to the kitchen around 7 a.m.. In keeping with the HELP-ME trial protocol, based on his symptoms and new ECG changes (panel B) compared to discharge (panel A) on his home ECG device, he was directed to the closest emergency department for further evaluation. Shortly after arrival to the emergency department he had a cardiac arrest due to ventricular fibrillation, was successfully resuscitated and admitted. Repeat coronary angiogram was again negative for any coronary culprit lesion; cardiac magnetic resonance imaging this time though was indicative of myocarditis.
